# Habitat prevails over host sex in influencing mycobiome structure of terrestrial isopod, *Armadillidium vulgare*

**DOI:** 10.1128/spectrum.02172-24

**Published:** 2025-03-31

**Authors:** Jiho Yang, Yehyeon Cha, Seung-Yoon Oh

**Affiliations:** 1 Gyeongnam Bio and Anti-aging Core Facility Center, Changwon National University 34925, Changwon, South Korea; 2 Department of Biology and Microbiology, Changwon National University, Changwon, South Korea; 3 Department of Biology and Chemistry, Changwon National University 34925, Changwon, South Korea; Barnard College, New York, New York, USA

**Keywords:** mycobiome, community structure, diversity, co-occurrence network, terrestrial isopod, *Armadillidium vulgare*

## Abstract

The terrestrial isopod, a crustacean order that has successfully transitioned from aquatic to land ecosystem, functions as a soil bioengineer and plays a crucial role in ecological decomposition. While there has been comprehensive documentation of bacterial associations with isopods, suggesting their contribution to digestive processes, the fungal component of isopods remains a less-explored dimension. Expanding our fundamental exploration into terrestrial isopods, we investigated whether the fungal community in *Armadillidium vulgare* is more significantly influenced by host sex or habitat using high-throughput sequencing-based internal transcribed spacer region amplification. Our findings revealed that the isopod mycobiome structure and taxonomic composition are predominantly shaped by the host’s habitat rather than its sex. While the fungal richness of the mycobiome varied based on isopod sex, the co-occurrence network exhibited distinct variations dependent on both habitat and host sex. Finally, we observed a complete overlap of core mycobiomes based on isopod sex and identified fungal residents that are consistently distributed in a manner specific to habitat. In conclusion, we assert that community assembly of the isopod mycobiome in an untouched ecosystem is more significantly influenced by location than sex. We contend that this offers a fundamental groundwork, laying the cornerstone for future investigations into terrestrial isopods—pivotal agents in ecological decomposition within ecosystems.

**IMPORTANCE:**

This study addresses a significant knowledge gap in the mycobiome of terrestrial isopods, an area that has received limited scientific attention despite extensive research on bacterial associations within these organisms. Using high-throughput sequencing, this study demonstrates that the habitat of *Armadillidium vulgare* exerts a more pronounced influence on the composition of its mycobiome compared with host sex. By examining variations in community structure, diversity, co-occurrence patterns, and identifying core mycobiomes and specialist taxa based on isopod location, this study provides crucial foundational data. These findings are essential for advancing future research on the ecological and evolutionary dynamics of fungal communities in terrestrial isopods.

## INTRODUCTION

Isopods, an order of crustaceans boasting a diverse array of over 10,000 recognized species, have effectively transitioned from aquatic to terrestrial realms, showcasing impressive ecological adaptability (1–3). A primary hurdle faced by these land-dwelling isopods is the conservation of water, a challenge stemming from the absence of typical cuticular lipids found in many other terrestrial arthropods (4, 5). To surmount this obstacle, isopods have developed distinctive physiological adjustments, including gill-like pleopods, vital for their survival and distribution across terrestrial landscapes (6, 7). Adaptations for water conservation in the body, coupled with their dietary preferences, may elucidate why terrestrial isopods prefer microhabitats characterized by temperate conditions and moderate humidity, such as the leaf litter (8). Terrestrial isopods not only consume organic constituents originating from plant sources but also feed on a variety of organisms such as lichens, algae, and occasionally dead insects (8–11). The digestive characteristics of terrestrial isopods suggest that they contribute directly and indirectly to decomposition processes in ecosystems (12, 13). The digestion of cellulose during gut passage (14, 15) results in lower levels of recalcitrant compounds in isopod feces compared with leaf litter (8). Additionally, through phenol degradation facilitated by isopods or their intestinal microbiota (13, 16), these organisms play a significant role in soil development (17).

The intricate interactions between microorganisms and macroorganisms have become a focal point in ecological research, unraveling the complex web of associations that govern terrestrial ecosystems (18, 19). Within this framework, the microbiome plays a pivotal role in shaping the ecological dynamics of various organisms (20–22). Following the initial identification of bacteria in the midgut glands of terrestrial isopods (23), there has been thorough documentation of bacterial associations. The suggested role of bacterial symbionts within the hepatopancreatic lumen is their potential contribution to the digestive processes of isopods. This includes activities, such as cellulose hydrolysis, lignin oxidation, and phenolic oxidation (8, 13, 16). In addition to bacteria associated with the digestive and decomposition processes of isopods, the following research has been conducted on a specific microbe, *Wolbachia pipientis. Wolbachia*, recognized for its capacity to transform male embryos into functional females (24, 25), has been identified as an obligate intracellular Alphaproteobacteria in *Armadillidium vulgare* (22). The specific molecular mechanism behind feminization in isopods remains elusive, but it is believed to involve the prevention of androgenic gland differentiation, the tissue responsible for synthesizing the androgenic hormone (26, 27). Recently, the overall composition of the isopod microbiome structure was unveiled using high-throughput sequencing technology, initially pioneered by Horvathova et al. (28), particularly in *Porcellio scaber*. This approach revealed that the primary dietary source predominantly shapes the taxonomic composition of the bacterial community in the isopod gut. Several studies have indicated that Proteobacteria and Bacteroidetes constitute the predominant phyla in the isopod microbiome (22, 29). Moreover, Wenzel et al. have asserted that the isopod microbiome is subject to influences in the order of seasonality, geography, and sex (29). It has been reported that the number of microbiome phyla present in the isopod microbiome is similar across species, yet their composition differs (30). Additionally, studies have shown that the abundance of specific microbial residents can vary significantly by sex (31, 32), and that diet influences taxonomic composition (33). However, to date, no research has simultaneously examined the impact of both sex and location on microbiome structure to determine which of these factors has a greater influence.

The fungal component of isopod presents an intriguing and less-explored dimension. Fungi are integral players in nutrient cycling (34, 35), plant–microbe interactions (36–38), and the decomposition of organic matter (39, 40). Understanding the mycobiome associated with isopods is crucial for comprehending the multifaceted roles these isopods play in ecosystem functioning. Despite the recognized importance of fungi in terrestrial ecosystems, only a limited number of studies have investigated the mycobiome of isopods. The application of a culture-dependent approach unveiled the existence of fungal residents, including *Baltomyces* and *Palaviascia*, in the gut of isopods (41, 42). While studies at the mesocosm scale have explored the ecologically positive effects of isopods, including the augmentation of methane uptake, modulation of bacteria–fungi interactions (43), and the suppression of dominant cord-forming basidiomycete fungi to enhance fungal diversity (33), the mycobiome within isopods remains relatively unexplored. In the pioneering research conducted by Heděnec et al. on the mycobiome of two isopod species, it was discovered that litter quality significantly influences feeding preferences, consumption rates, and composition of fungal communities in the gut and feces of *Oniscus* and *Glomeris* (44). Subsequently, Cha and Oh utilized metabarcoding to compare fungal diversity of two isopod species, *Armadillidium nasatum* and *A. vulgare* (45). Results showed *A. nasatum* with significantly higher mycobiome alpha diversity than *A. vulgare*. Subsequently, they also discovered that terrestrial isopods, such as *Porcellio laevis*, serve as habitats for diverse fungal residents including *Stereum* and *Penicillium* (46). To the best of our knowledge, these studies are the sole endeavors to investigate the isopod mycobiome using high-throughput sequencing, with no previous examinations documented in the current literature. This research gap presents an opportunity to expand our understanding of the ecological dynamics within isopod microhabitats and the potential implications for broader ecosystem processes. Fundamental investigations are essential to understand how the isopod mycobiome is influenced by biotic and abiotic factors, such as host sex and habitat. Moreover, the recognition and interaction between the gut fungal community and the mucosal immune system are vital for sustaining a harmless relationship (47). A lack of immune mechanisms for recognizing fungi can lead to fungal overgrowth and systemic diseases in animals (48, 49). Furthermore, exposure to environmental fungi may be essential for developing a stronger immune response (50). Hence, investigating the mycobiome of terrestrial isopods, which are ecologically important, is crucial.

*Armadillidium vulgare*, the widely known terrestrial isopod crustacean with a global distribution, has long been regarded as a beneficial organism in ecosystems (51, 52). The foraging activities of *A. vulgare* expedite the decomposition process, aiding in the restoration of essential nutrients to the soil (10, 53, 54). In broadening the horizon of our fundamental exploration into terrestrial isopods, we delved into determining whether the fungal community in *A. vulgare* is more significantly influenced by host sex or habitat. In the previous study (28), we noted that the isopod microbiome varies depending on diet. Based on this, we hypothesized that location differences would have a greater impact on the isopod mycobiome than sex. This was achieved through the application of high-throughput sequencing-based internal transcribed spacer (ITS) region amplification. Employing multidimensional statistical analyses, we discerned factors that wield a more pronounced impact on the mycobiome. Furthermore, we investigated the variations in community structure, diversity, co-occurrence patterns, and the list of core mycobiome and specialists in accordance with each variable. We consider the value of our research lies in unveiling the mechanisms by which fungal residents within terrestrial isopods shape a community assembly in an untouched natural ecosystem.

## MATERIALS AND METHODS

### Isopod sampling and DNA extraction

We collected the isopod samples from four locations of urban parks in South Korea: Changwon (Site 1, 35°18′23.50″N, 128°41′21.03″E), Gimhae (Site 2, 35°18′35.69″N, 128°43′3.86″E), Miryang (Site 3, 35°22′37.76″N, 128°42′30.93″E), Changnyeong (Site 4, 35°22′40.79″N, 128°38′19.60″E). The vegetation is various depending on the locations, but the conifers (e.g., *Pinus* spp.) and broad-leaved trees (e.g., *Prunus* sp., *Rhododendron* sp., and *Zelkova serrata*) are mixed. The four collection sites are relatively close (ca. 5 km) to each other and were targeted for simultaneous sampling in areas with abundant leaf litter, where isopods are frequently found during the spring season. Although microclimate data are not available, it is reasonable to assume that the conditions are similar across these sites. From each location, a total of six individuals, three females and three males, were found and collected under the litters and stones. The species and sex of isopod were identified morphologically using a stereoscopic microscope Leica MZ10 F (Leica Biosystems, Nußloch, Germany).

The surface of the isopod samples underwent sterilization using 70% ethanol for 1 min, followed by washing with sterilized distilled water for another minute to eliminate soil and plant debris. Post-sterilization, the distilled water utilized for washing (100 µL × 3 replicates) was inoculated onto a 90 mm PDA medium (BD Difco, Franklin Lakes, NJ, USA) to validate the surface sterilization status of the samples. After incubation at 25°C for 2 weeks, it was verified that no microorganisms grew on the medium. To confirm the death of the samples, they were placed in a freezer (−20°C) for 5 minute. The sample, weighing approximately 0.05 g, was homogenized in a 2 mL tube with a steel bead using a Taco Prep Bead Beater (GeneReach, Taichung, Taiwan) for 13 s. Genomic DNA was extracted from the crushed samples using an AccuPrep DNA extraction kit according to the manufacturer’s protocol (Bioneer, Daejeon, South Korea). The DNA was stored at −20°C until further use.

### Molecular experiments

For the Illumina MiSeq preparation, the fungal ribosomal ITS region was amplified in two steps using an AccuPower PCR PreMix kit (Bioneer, South Korea). Initially, the full ITS region was amplified with ITS1Fngs and ITS4 primers (55, 56) under the following conditions: 95°C for 5 min, 35 cycles of 94°C for 30 s, 55°C for 30 s, 72°C for 40 s, and 72°C for 5 min. The amplicons were confirmed through 1% gel electrophoresis and used for the secondary PCR amplification of the ITS1 region. The primers ITS1Fngs and ITS2ngs (57) were attached to the MiSeq adapter, and the thermocycling conditions were as follows: 95°C for 5 min, 15 cycles of 95°C for 30 s, 55°C for 30 s, 72°C for 40 s, and 72°C for 7 min. The amplicons were again confirmed through 1% gel electrophoresis and then purified using an Expin PCR-SV kit (GeneALL, Seoul, South Korea). PCR was conducted in triplicate for each sample using a SimpliAmp Thermal Cycler (Applied Biosystems, Waltham, MA, USA), and the results were pooled after measuring the DNA quantity using a Multiskan SkyHigh Microplate Spectrophotometer (Thermo Fisher Scientific, Waltham, MA, USA). Sequencing was performed using Illumina MiSeq at Macrogen (Seoul, South Korea). All sequences generated in this study were deposited to NCBI Sequence Read Archive under BioProject ID PRJNA1068791.

### Bioinformatics and statistics

The initial processing of raw sequencing data involved utilizing the QIIME2 platform (58). Following de-multiplexing and adapter trimming, sequence pairs underwent denoising and merging through the application of DADA2 (59). VSearch was employed to perform operational taxonomic unit (OTU) clustering, considering a 97% sequence similarity threshold, and the removal of chimeric sequences was carried out (60). Representative sequences for each OTU were taxonomically assigned using the Naïve Bayesian classifier (61) against the NCBI RefSeq database (62). To maintain consistency across all analyses except for those described in the following sentence, all samples were normalized to the lowest sequence count, set at 35,000 reads (the lowest: 35,973). Beta diversity analysis, ordination, and differential abundance methods were performed using non-rarefied compositional data (63).

Subsequent analyses were conducted using R software version 3.5.3 (64). Prior to statistical comparisons, the normality of the data were assessed through the Shapiro–Wilk test (65). Given the violation of the normality assumption (*P* < 0.05), non-parametric tests were employed, specifically the Kruskal–Wallis test (66) followed by the Bonferroni correction (67) for multiple comparisons and the Mann–Whitney U test (68) for pairwise comparisons. We performed the following analyses based on Aitchison distance for assessing the beta diversity of the fungal community (69). Principal component analysis (PCA) was utilized as a dimensionality reduction technique to analyze data variance through the “microbiome” package, and the statistical examination of whether the isopod location and sex exert an influence on mycobiome beta diversity was conducted through permutational multivariate analysis of variance (perMANOVA) using the “microbial” package (70, 71). Dissimilarity comparisons between the variables of sex and location involved extracting dissimilarity matrices from samples with different sexes within the same location and those with different locations but the same sex. Variation partitioning analysis was carried out utilizing the “vegan” package. The identification of the top 50 OTUs was based on average relative abundance, and a hierarchical heatmap was visualized using the “pheatmap” package (72). The OTU co-occurrence network was implemented through the “seqtime” and “SpeicEasi” packages (73, 74). The core mycobiome for each isopod sex was identified using the “microbiome” package with a prevalence threshold of 0.5. OTUs exhibiting different frequency distributions concerning isopod sex were analyzed using the Analysis of Compositions of Microbiomes with Bias Correction (ANCOM-BC) method (75). Linear discriminant analysis (LDA) was employed via the “microbial” package to identify isopod specialist fungal residents specific to different locations, with OTUs having LDA score >2 and a *P* value of < 0.05.

## RESULTS

### Isopod mycobiome structure is predominantly shaped by the host’s habitat rather than its sex

**Fig 1:**
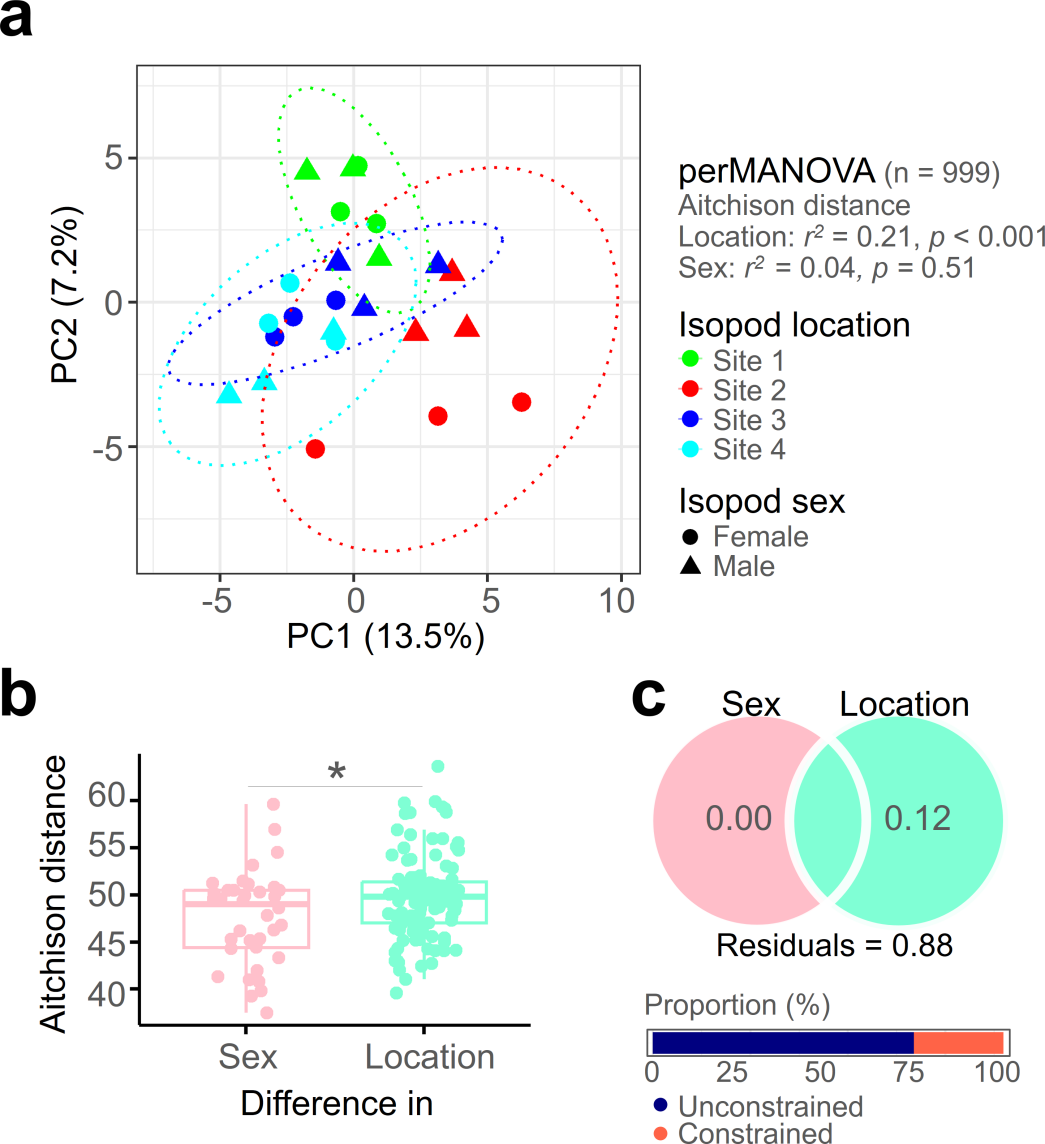
The mycobiome structure of isopods is predominantly influenced by the host’s habitat rather than its sex. The isopod mycobiome is visualized through principal component analysis after dimensionality reduction, with ellipses indicating 95% confidence intervals (**a**). Aitchison distance differences based on various variables are compared between isopod mycobiomes of different sexes within the same habitat and those of the same sex in different habitats (**b**). The partitioning variable is visualized through a Venn diagram, and numerical values are rounded to two decimal places (**d**). * *P* < 0.05.

In an attempt to simplify the visualization of relationships between communities, we employed dimensionality reduction (Fig. 1a). The PCA ordination indicates that communities seem to be more influenced by the location of the host than the host’s sex. However, clustering by sex within the same location was found, except for Site 1. The perMANOVA (*n* = 999) results indicate that the location (*r*^2^ = 0.21, *P* = 0.001), rather than the isopod sex (*r*^2^ = 0.04, *P* = 0.51), significantly influences the mycobiome structure. For a clearer understanding of the influence of the two variables on the mycobiome, we attempted a statistical approach (Fig. 1b). Aitchison distance based on sex within the same location was lower than the mycobiome dissimilarity within the same sex inhabiting different locations (Mann–Whitney, *P* < 0.05). This suggests that the isopod mycobiome is significantly more influenced by the habitat than the host’s sex. Confirming this, variable partitioning showed that location has a more substantial impact on the mycobiome (Fig. 1c). The high unconstrained proportion in the analysis results (76%) indicates that there is still a considerable amount of variability not accounted for by the current explanatory variables. In summary, the community structure of the isopod mycobiome is significantly influenced by the tested variables, host sex, and more prominently, the collection site.

### Fungal richness in isopod mycobiome shows distinct variations contingent on both habitat and host sex

**Fig 2:**
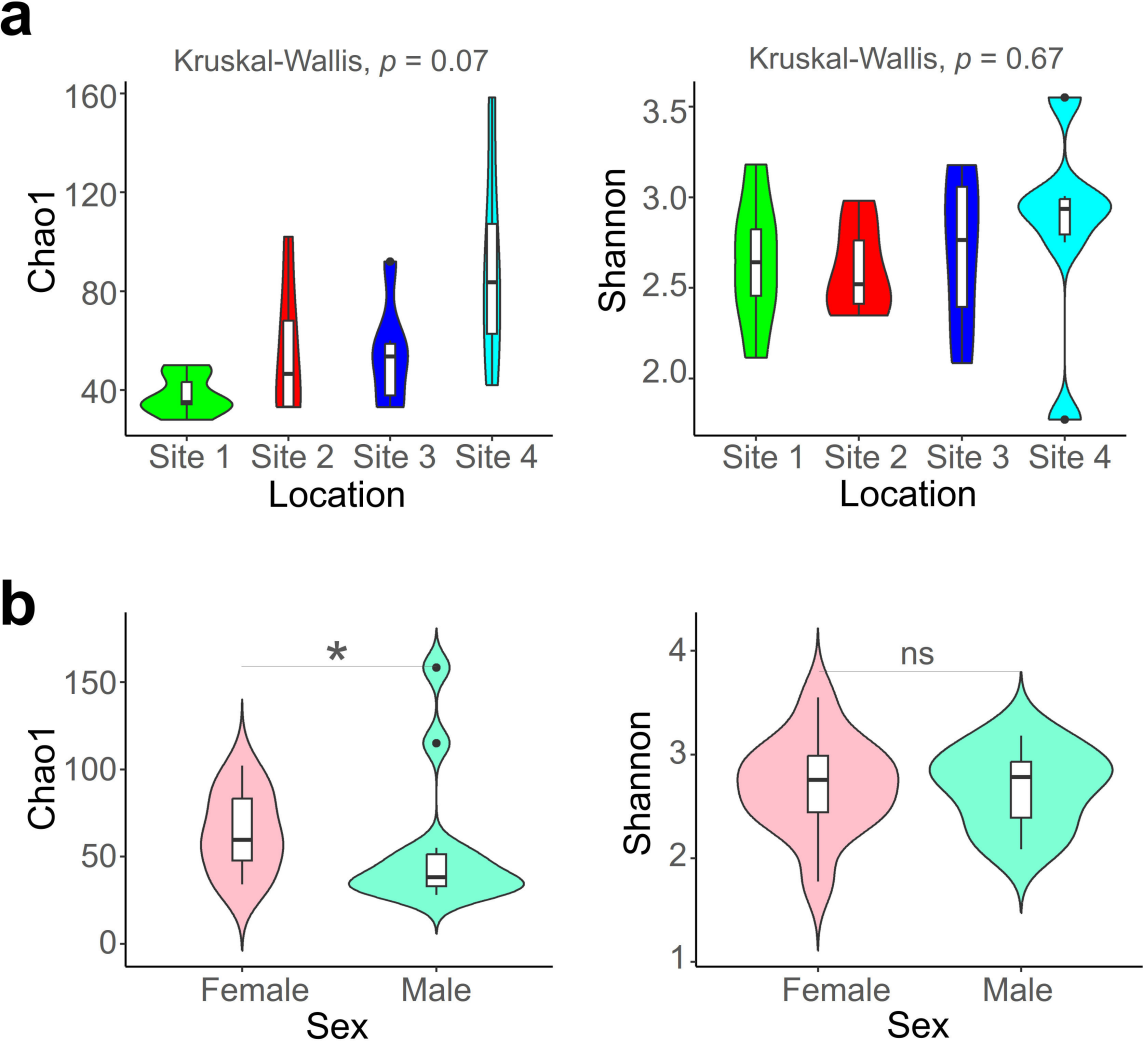
Within the isopod mycobiome, fungal richness exhibits discernible variations influenced by both habitat and host sex. The alpha diversity of the isopod mycobiome, represented by Chao1 for species richness and Shannon for species diversity, is compared across habitats (**a**) and sex categories (**b**). * *P* < 0.05, ns, not significant.

The investigation into the alpha diversity of the isopod-associated fungal community, concerning host sex and habitat, revealed interesting patterns. In contrast to the substantial influence of location on community structure observed in previous analyses, alpha diversity did not vary statistically across locations (Fig. 2a). Chao1, representing species richness, peaked at Site 4, followed by Sites 3, 2, and 1, with no statistically significant differences among the four groups (Kruskal–Wallis, *P* > 0.05). The Shannon index, representing species diversity, did not exhibit statistically significant differences across the four locations (Kruskal–Wallis, *P* > 0.05). Unexpectedly, Chao1 displayed a significant difference based on the sex of isopods (Fig. 2b). The species richness of the mycobiome associated with female isopods was higher than that of male isopods (Mann–Whitney, *P* < 0.05). However, species diversity (Shannon) did not differ significantly based on sex. In summary, significant variations in the alpha diversity of the isopod mycobiome were observed based on host sex.

### The taxonomic makeup of the isopod mycobiome is significantly dictated by location rather than sex of host

**Fig 3:**
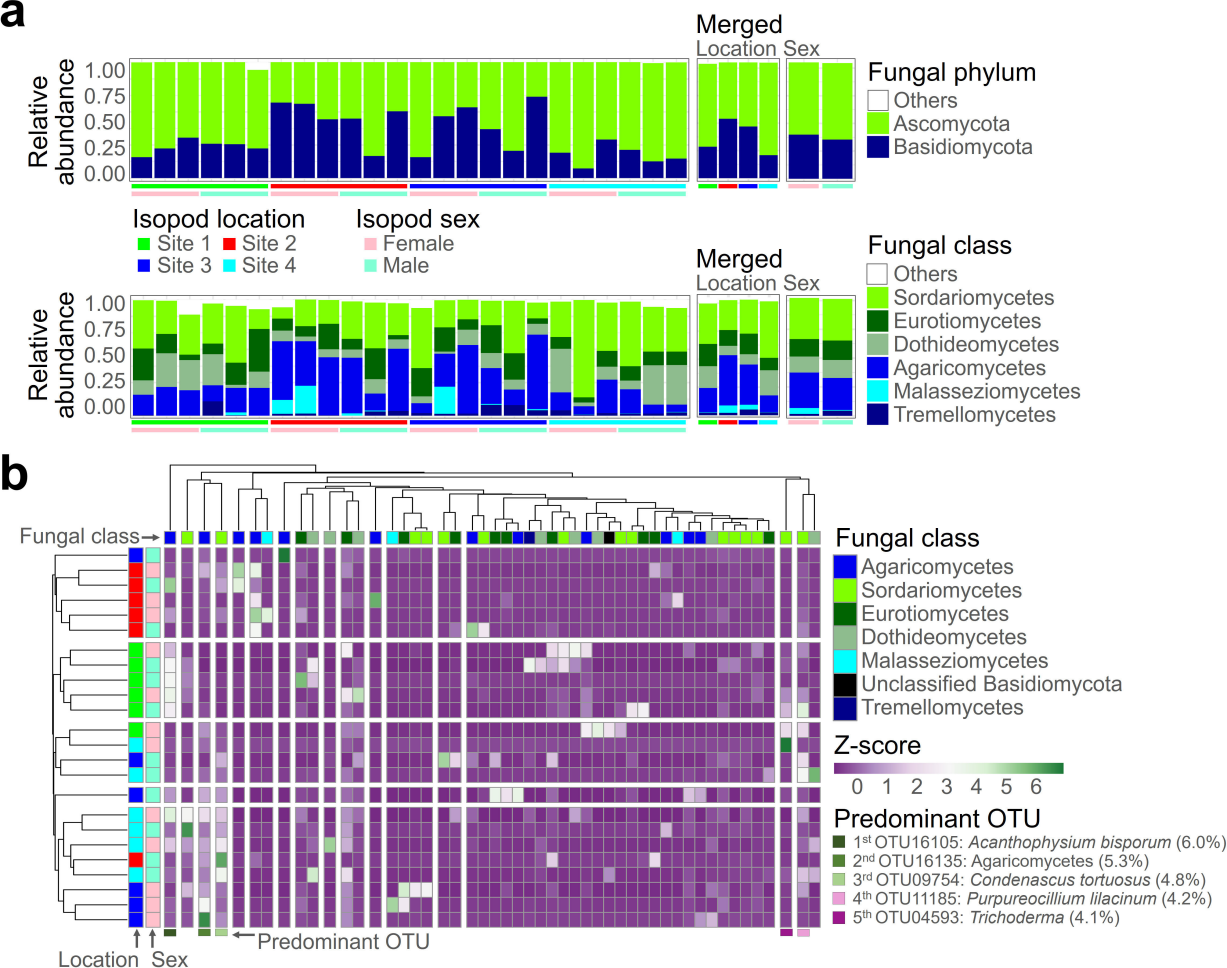
The taxonomic composition of the isopod mycobiome is prominently influenced by the host’s location rather than its sex. The abundance of each taxon within the isopod mycobiome is visually represented as relative frequencies at the fungal phylum and class levels (**a**). Merged plots factoring in two variables, location and sex, are displayed on the right. The distribution patterns of the top 50 abundant operational taxonomic units (OTUs) are depicted using a hierarchical heatmap (**b**). Columns represent fungal OTU taxa, while rows incorporate information on the host isopod’s location and sex. The fungal distribution is portrayed in z-scores, with predominant OTUs identified as the top five OTUs with the highest mean relative frequencies.

In an overarching perspective, particularly when examining the phylum level, the fungal community was predominantly represented by Ascomycota (64%), followed by Basidiomycota (36%) (Fig. 3a). When examining the class level, Sordariomycetes dominated within the Ascomycota phylum (34%), followed by Eurotiomycetes (16%) and Dothideomycetes (15%). Within the Basidiomycota phylum, Agaricomycetes (29%) took the lead, followed by Malasseziomycetes (3%) and Tremellomycetes (3%). As evident in the merged plot, the composition of isopod mycobiome varied significantly according to the habitat. While Ascomycota generally dominated, the degree of dominance was pronounced in Sites 1 and 4, whereas Basidiomycota showed higher relative frequencies in Sites 2 and 3. The variation in the taxonomic composition of the fungal community across locations was attributed to the higher relative distribution of Dothideomycetes in Sites 1 and 4, and the increased proportion of Agaricomycetes in Sites 2 and 3 (considering Sites 2 and 3, Agaricomycetes was the most dominant taxa at the class level, 38%). In contrast, there was no pronounced taxonomic difference in the mycobiome based on host sex, except for a slightly higher relative frequency of Malasseziomycetes within the Basidiomycota in the female mycobiome. In summary, the taxonomic composition of the isopod mycobiome was significantly influenced by the location rather than the host sex.

Supporting evidence for the differentiation of the isopod mycobiome based on habitat rather than host sex is illustrated in the hierarchical heatmap of the top 50 abundant OTUs (Fig. 3b). The distribution patterns of fungal residents in Sites 1 and 2 are completely distinct from those in other sites, while Sites 3 and 4, to a lesser extent, exhibit noticeable clustering based on location differences. Identifying five predominant OTUs based on mean relative abundance, *Acanthophysium bisporum* emerges as the most abundant (6.0%), particularly prevalent in Site 1. Following this, unidentified Agaricomycetes (5.3%), *Condenascus tortuosus* (4.8%), *Purpureocillium lilacinum* (4.2%), and *Trichoderma* (4.1%) rank subsequently.

### Isopod mycobiome’s co-occurrence network pattern showcases variations contingent on both host sex and habitat

**Fig 4:**
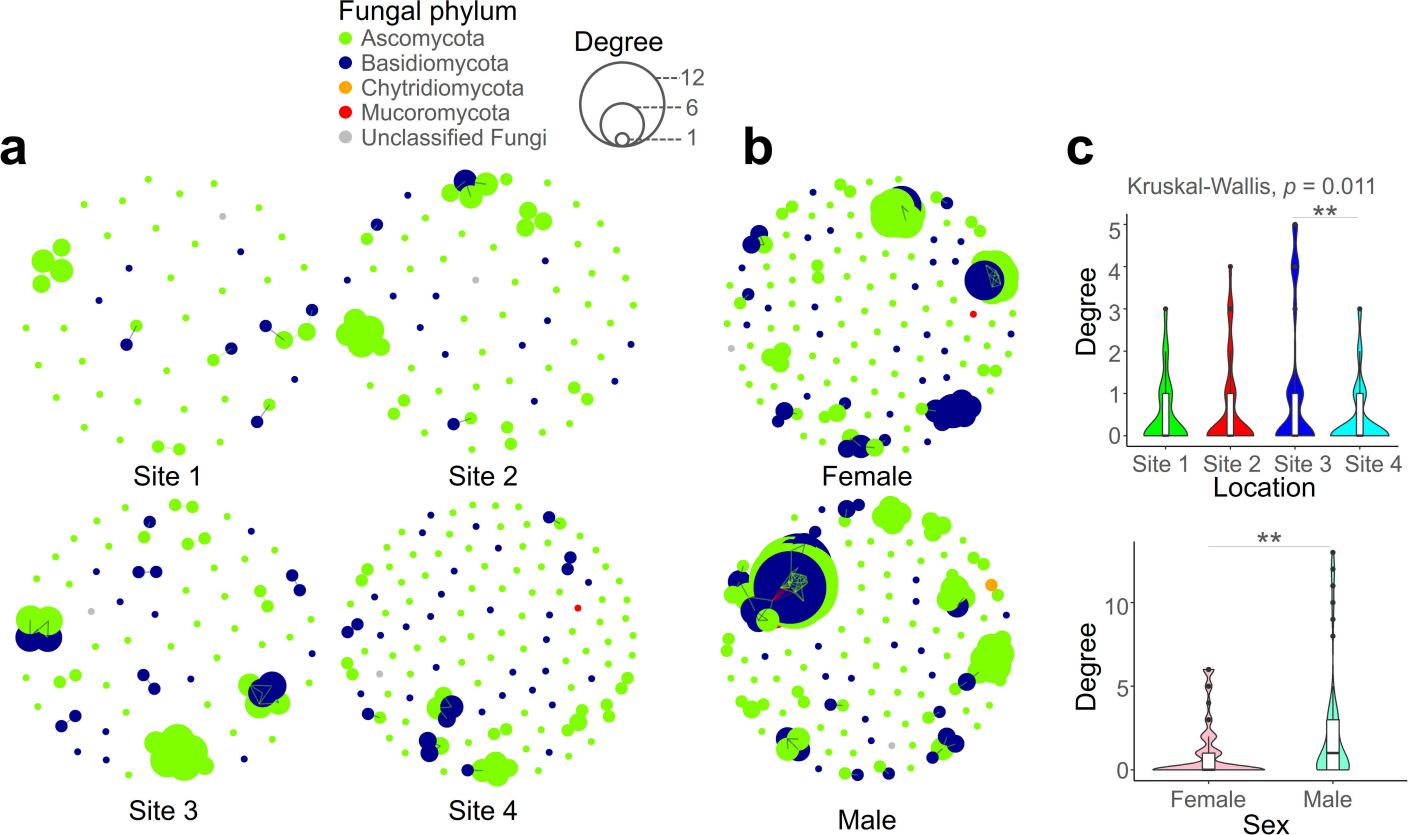
Co-occurrence network pattern of isopod mycobiome exhibits distinct variations contingent upon both sex of host and its habitat. Visualization of the co-occurrence patterns of the isopod mycobiome is presented through network plots, distinguished by habitat (**a**) and sex (**b**). The color of nodes corresponds to the phylum of fungal OTUs, while the size of nodes is indicative of the degree, representing the magnitude of correlations between adjacent nodes. Statistical comparisons of degree differences of the isopod mycobiome based on habitat and sex were assessed (**c**). ** *P* < 0.01.

Examining the co-occurrence patterns among OTUs within the fungal community associated with isopods, we visualized how these patterns differ based on habitat (Fig. 4a) and host sex (Fig. 4b). In the site-wise comparison, the degree, representing the number of edges held by a single node, varied significantly across sites (Fig. 4c, Kruskal–Wallis, *P* < 0.05). The fungal community located in Site 3 exhibited the highest degree, and a notable difference was observed in the higher co-occurrence of Basidiomycota compared with other sites. The co-occurrence tendencies of the isopod mycobiome were significantly influenced by host sex (Fig. 4c, Mann–Whitney, *P* < 0.01). The degree was higher within male samples, suggesting that the mycobiome inside male isopods possesses a more robust structure than that of females.

### Identification of core mycobiome specific to isopod sex and fungal residents that are frequently distributed in a habitat- or host sex-specific manner

**Fig 5:**
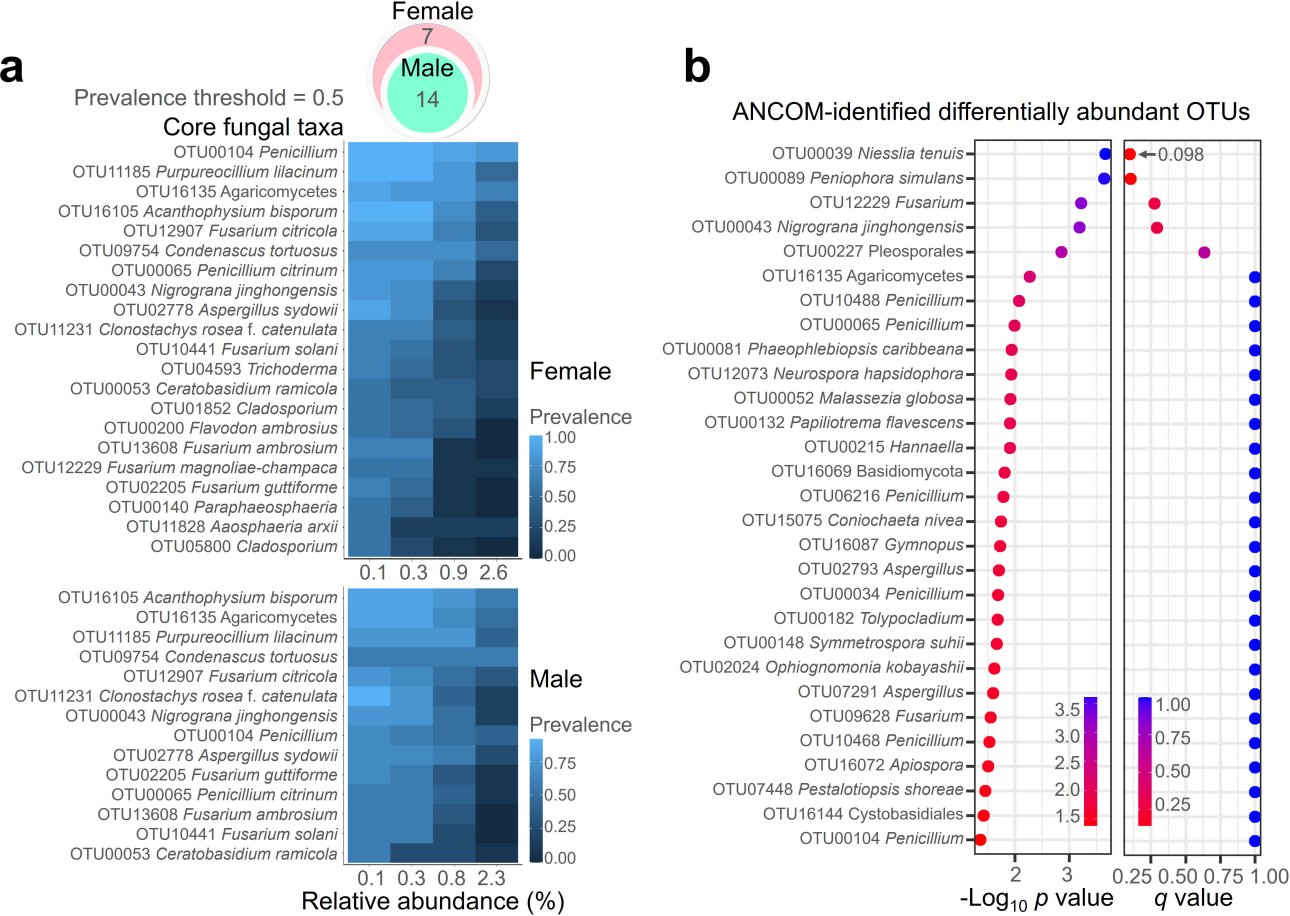
Recognition of core mycobiomes and fungal residents with distinct distribution patterns linked to isopod sex. Core mycobiomes based on isopod sex were identified with a prevalence cutoff of 0.5 (**a**). The changes in prevalence according to the average relative abundance variation were visualized using a heatmap, while a Venn diagram depicted the pattern of overlap between female and male core mycobiomes. Within the isopod mycobiome, OTUs showcasing disparate distributions associated with host sex were discerned using the ANCOM-BC method (**b**).

In order to ascertain which fungal residents are predominantly associated with isopod sex, we identified core mycobiomes based on a prevalence threshold of 0.5 (Fig. 5a). The higher fungal richness observed in female isopods corresponded to a greater number of core mycobiomes in females (21) compared with males (14). Notably, two core mycobiomes exhibited complete overlap. The core mycobiome included all five predominant OTUs identified, such as *Acanthophysium bisporum*, Agaricomycetes, and *Condenascus tortuosus*. ANCOM identified differentially abundant OTUs based on isopod sex. A total of 29 OTUs had a *P*-value below 0.05; however, their corresponding q-values were all above 0.05 (Fig. 5b). This suggests that there are no fungal OTUs with significantly different abundance between isopod sexes.

**Fig 6:**
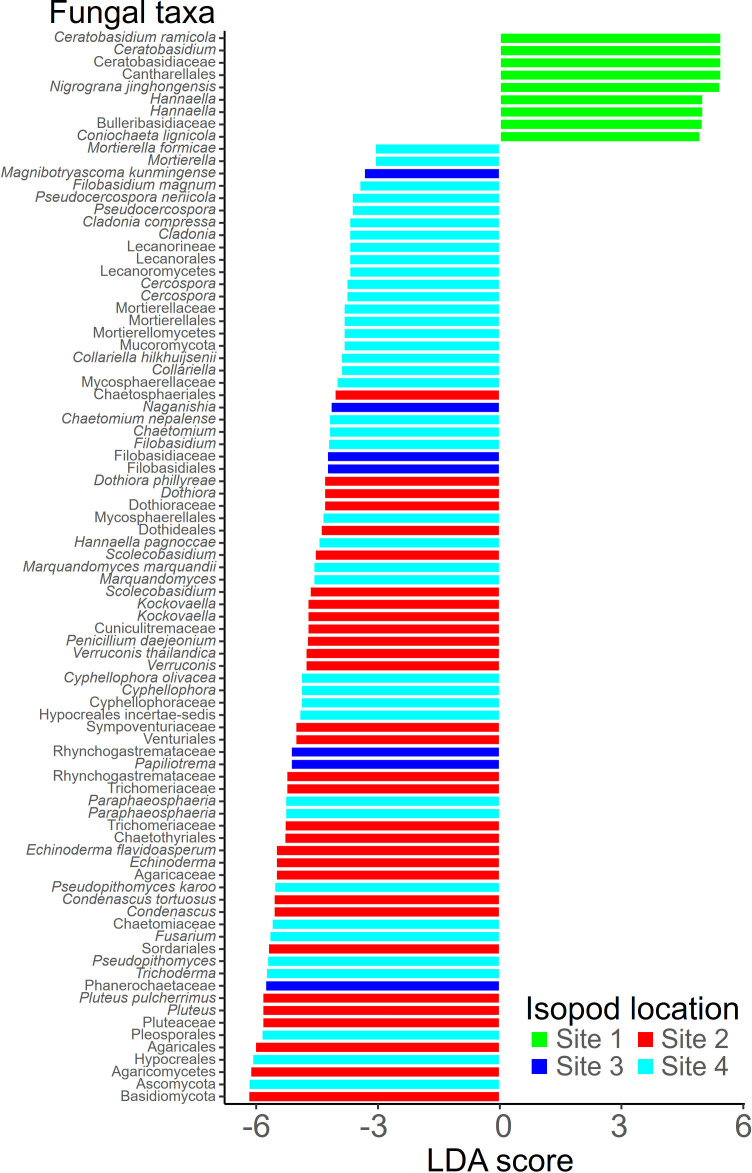
Fungal communities exhibiting varied distributions in relation to habitat. Utilizing linear discriminant analysis (LDA), a statistically significant roster of fungal specialists unique to each habitat was obtained, adhering to criteria including an LDA score >2 and a *P*-value < 0.05.

Through linear discriminant analysis (LDA), we obtained a statistically significant list of fungal specialists specific to each habitat based on the criteria of LDA score >2 and *P* value < 0.05 (Fig. 6). Among the OTUs taxonomically assigned at the genus level or below, species, such as *Ceratobasidium ramicola* and *Nigrograna jinghongensis*, were identified as notably and frequently distributed in Site 1. In Site 2, *Pluteus pulcherrimus* and *Condenascus tortuosus* were observed to occur at higher frequencies compared to other regions, while Site 3 exhibited abundance of *Papiliotrema* and *Naganishia*. In Site 4, the isopod mycobiome revealed a higher distribution of species, such as *Collariella hilkhuijsenii* and *Cercospora*, compared with other regions. Notably, *Cladonia compressa*, known as a lichen, was also identified as a specialist in this region.

## DISCUSSION

Functioning as ecological decomposers, terrestrial isopods are recognized for occupying a vital niche in ecosystems, contributing to soil fertility through material cycling. Recently, there has been a heightened emphasis on understanding the impact of microbiomes on their hosts or environments, leading to active research on the isopod microbiome. While bacterial communities within isopods have garnered attention, the less-explored fungal residents prompt us to fortify our foundational knowledge. As a result, our investigation aimed to discern whether the isopod mycobiome is more profoundly influenced by host sex or location, contributing to the advancement of fundamental insights in this domain. We have uncovered the following observations and aim to discuss them. The isopod mycobiome structure and taxonomic composition are primarily influenced by the host’s habitat rather than its sex. Fungal richness and co-occurrence network pattern in the isopod mycobiome exhibit discernible differences dependent on both habitat and host sex. Finally, we have identified fungal residents that exhibit frequent distribution patterns specific to either the habitat or host sex.

Significant effects arising from variations in location on the isopod mycobiome structure have been observed, surpassing the impact of host sex. The lack of specific data, such as soil chemical properties, vegetation, and other habitat-related factors, prevents a precise determination of the actual variables affecting the isopod mycobiome. Nevertheless, this pattern mirrors earlier findings where isopod-associated bacterial communities were more significantly influenced by location differences than host sex (29). Given the geographical proximity and minimal climatic disparity among the four collection sites for isopods, it is plausible that variations in prey composition within each habitat contributed to these outcomes. This inference is supported by previous research demonstrating that differences in the ingestion of feed with varying lignin and cellulose compositions resulted in distinctions in the isopod gut and feces microbiome structure (44). Notably, fungal residents belonging to *Acanthophysium* and *Trichoderma* remained predominant in the isopod mycobiome, irrespective of location. This aligns with prior research identifying these fungi as dominant taxa within *Armadillidium vulgare* (45). The apparent abundance of these fungi in isopods, known for their consumption of plant materials and organic layers (8, 16), is likely associated with the diverse repertoire of carbohydrate-active enzymes exhibited by the fungi (76, 77).

The isopod sex exhibited a nuanced influence on the internal mycobiome, with male-associated fungal communities suggesting a lower species richness and higher co-occurrence, indicative of a nested network in males compared to females. This observation contrasts with previous research indicating higher microbiome diversity in males of intertidal isopod *Jaera* compared to females (29). In *Armadillidium vulgare*, sex determination is known to be governed by both sex chromosome heterogamety and homogamety, alongside feminizing actions mediated by *Wolbachia* and intracytoplasmic factors (78). Behavioral differences associated with sex are evident during the breeding season, where *A. vulgare* females, carrying eggs and newly hatched young in a brood pouch, exhibit a considerably smaller activity radius compared with males (10). Survival capabilities under humidity stress revealed that *A. vulgare* females exhibited lower survival rates than males, and their response to stress manifested through an increased frequency of volvation (79). Furthermore, physiological biomarkers of aging, such as beta-galactosidase activity and weak cell viability, were more prominently expressed in aged *A. vulgare* females compared with males (80). Interestingly, the core mycobiomes specific to isopod sex exhibited complete overlap. This implies the existence of a robust fungal group firmly established within *A. vulgare*, irrespective of host sex. Additionally, the use of ANCOM-BC revealed that there are no differentially abundant OTUs based on isopod sex, which aligns with the observation that a significant portion of the core mycobiome overlaps across sexes. This suggests that the isopod mycobiome is notably conserved regardless of sex in this study.

While this study offers fundamental insights into the previously understudied fungal community of the globally distributed *Armadillidium vulgare*, it has some limitations. Specifically, it demonstrates that the mycobiome is significantly affected by location differences but does not clearly define specific factors—such as diet, challenges in generalization due to insufficient sample size, interactions with the environment, seasonal effects, and others—that contribute meaningfully to this variation. The utilization of whole *A. vulgare* individuals as metabarcoding samples, without specifically targeting the gut or feces, further obscures the identity of this mycobiome. Despite these limitations, we consider our study valuable as it lays the foundation for understanding how the *A. vulgare* mycobiome community assembly occurs within natural ecosystems that are not manipulated. Future endeavors, involving more refined sampling strategies and research designs, are anticipated to offer a clearer depiction of the terrestrial isopod’s mycobiome within the ecosystem, elucidating their key niches and roles.
